# Supplementary material for: Are carbon emissions trading and green financial instruments synergistic? -Comprehensive quantitative research based on content analysis
Source: PLoS One. 2024 Mar 7;19(3):e0298601. doi: 10.1371/journal.pone.0298601 (PMC10919601; doi:10.1371/journal.pone.0298601)
Supplement: S1 Data — (ZIP) [file pone.0298601.s001.zip › Data/地级市Z_F工作报告.zip!/╔╜╢1⁄2/╚╒╒╒╩╨/└┤╫╘╣1⁄2╓┌║┼í╛┬φ┐╦╩2╛▌═°í┐_2015.docx]

各位代表：

现在，我代表市人民政府向大会报告工作，请予审议，并请市政协委员提出意见。

一、2014年政府工作回顾

2014年，是我市在攻坚克难中奋力前行的一年。市政府紧紧依靠省委、省政府和市委的坚强领导，依靠市人大、市政协的监督支持，依靠全市人民的共同努力，深入贯彻党的十八大及十八届三中、四中全会和习近平总书记系列重要讲话精神，以开展党的群众路线教育实践活动为动力，抓改革、稳增长、调结构、强蓝区、优生态、惠民生、保稳定，全市经济健康稳定增长，社会事业全面进步，民生得到持续改善。

经济发展稳中有进。全年实现生产总值1611.87亿元，比上年增长10%，增幅居全省第3位。三次产业比例调整为8.6：50.4：41。实现公共财政预算收入111.07亿元、支出167.77亿元，分别增长11%和6%。现代农业持续健康发展。粮食产量连续10年稳定在100万吨以上，特色种植业面积突破140万亩，无公害农产品、绿色食品、有机农产品达到334个。现代渔业、畜牧业稳步发展。完成植树造林12.6万亩。各类新型农业经营主体发展到5406家。新建水利工程1282处。工业转型升级迈出新步伐。产值过百亿的企业发展到7家，新增2家，规模以上工业增加值增长10.9%。汽车及核心部件制造业完成产值521.72亿元，增长12.7%。日照钢铁精品基地完成投资76.1亿元。日钢高档薄板、大H型钢、森博液体包装纸、金禾博源高纯度柠檬酸等126个工业项目建成投产。服务业增加值增长12%，增速居全省首位，占GDP的比重提高2个百分点。社会消费品零售总额增长12.6%。完成港口货物吞吐量3.53亿吨，跃居全国沿海港口第8位；集装箱吞吐量在全国沿海港口提升2个位次。全市本外币贷款余额1887.57亿元，增速居全省之首。新增各类金融机构和新型金融组织21家。日照银行在全省地方商业银行绩效评价中名列第一。日照大宗商品交易中心组建运营。窝窝商城、微网、艾格拉斯、食乐淘等电商企业入驻日照。实现旅游总收入237.9亿元，增长13.2%。出台了大力促进民营经济加快发展的46条政策，全年民营经济增加值增长12.5%，占GDP的比重达到47.1%。

一批事关长远发展的大项目实现重大突破。围绕实施“双十•一城”工程，加大投资力度，完成固定资产投资1234.75亿元，增长15.5%；开工建设市级大项目87个，完成投资575.2亿元。瓦（塘）日（照）铁路建成通车，打通了我市向西开放、融入“一带一路”的新干线。日照港第2个30万吨级原油码头等6个泊位投入试运行，第3个30万吨级原油码头获得批复。胶新铁路电气化改造工程送电通车。龙潭沟水库实现下闸蓄水，“沭水东调”工程完成投资2亿元，3座中型水库除险加固和“北水南调”三期主体工程基本完成。220千伏双墩埠等15项输变电工程建成投运。特别令人振奋的是，经过积极努力，全市瞩目的日照民用机场可研报告获国家发改委批复，争取国家民航发展基金补助2.55亿元，航站楼和综合商务中心已封顶，土石方工程基本完成；石臼港区规划调整方案获交通运输部和省政府联合批复；青日连铁路开工建设，青日连铁路与济青高铁联络线获准与济青高铁同步兴建；鲁南城际铁路项目启动，日照至临沂段将与临沂至菏泽段同步建设。这些重大交通设施项目的上马，标志着日照的交通状况将实现历史性跨越，进一步构建起海陆空现代化综合交通运输体系。

“双轮驱动”取得新进展。一是全面深化改革实现新突破。去年是全面深化改革的第一年，我们按照市委总体部署，扎实推进重点领域改革，实现了开好局、起好步的目标。行政体制改革取得重要进展和成效，市级政府机构改革任务基本完成，行政审批项目减少到236项，精简下放率33.8%。商事制度改革强力推进，新增各类市场主体2.83万户，增长38.7%。金融综合改革成效明显，有4家企业在“新三板”挂牌，23家企业在区域性股权交易市场挂牌。财税体制改革全面推进，市县两级财政预决算、“三公”经费预决算、部门预决算全面公开，全市“三公”经费支出下降23.3%。农村综合改革持续推进，有65.5%的涉农村完成了农村土地承包经营权确权登记颁证，在59个村启动了农村集体经济组织股份制改革试点，有51个乡镇建立了农村产权交易平台；水利工程确权划界工作全面完成。社会保障体制改革迈出新步伐，城镇居民和农村居民基本养老保险、基本医疗保险实现并轨，在全省率先建立了居民医保与职工医保转换机制，出台了职工和城乡居民大病保险医疗救助政策。二是开放水平不断提高。抢抓“一带一路”国家战略机遇，我市被列为新亚欧大陆桥经济走廊主要节点城市，与国外7个城市建立了友城关系或签署友好合作意向书。日照港口岸获得国际联运过境货物运输业务资质，开通了至韩国釜山港集装箱班轮和至中亚集装箱跨境海铁联运、至蒙古海陆联运业务。晨曦集团直通式海关监管场站建成投运。成功举办了以“21世纪海上丝绸之路与海员服务发展”为主题的2014中国航海日论坛和旅韩华人华侨恳谈会。全年共招引推进投资5000万元以上的项目453个，实际到位资金322.5亿元。新批外商投资项目26个，实际到账外资5.73亿美元，增长8.1%。在国际市场需求持续回落的情况下，进出口总值增长5.2%，其中出口增长23.5%，增幅高于全省15.6个百分点。核准境外投资额2.74亿美元，增长108.3%，增幅居全省第2位。岚桥集团成功并购澳大利亚西部能源集团，浮来春集团在柬埔寨设立了日照产业园。三是创新驱动取得新成效。中国海洋人才市场（山东）日照分市场挂牌成立。成功举办了“高端外国专家日照行”活动，全年共引进外国专家及海内外高层次人才534名、创新创业团队15个。新增高技能人才6831人，日照技师学院被批准为国家级高技能人才培训基地。新创建省级以上创新平台13家。全年实现高新技术产业产值537.2亿元，增长21%，占规模以上工业总产值的比重提升1个百分点。

城乡建设和生态保护同步推进。中心城区完成城建投资108亿元，68公里山海路西延工程全线通车，中心商务区淄博路及地下建设完成工程量的80%；新增城市绿化面积120万平方米。拆迁改造城中村房屋3651处，建设安置房72万平方米。放宽进城落户门槛，新增城镇常住人口4.5万人，城镇化率达到52.71%，提高1.4个百分点。新增通自来水村105个、电气化村549个，改造农村公路312.6公里。“生态建设年”活动成效明显，十项环保重点治理工程基本完成，市区空气二氧化硫、二氧化氮、PM10、PM2.5浓度分别改善15.8%、10%、7.5%和10.6%，全年“蓝天白云、繁星闪烁”天数达到283天，保持在全省第一方阵。崮河、沭河等重点流域水质明显改善。我市海洋环境保护被国家海洋局作为全国唯一地级市项目，推荐申报全国生态保护与建设示范区。美丽文明乡村建设取得新成效，国家级生态乡镇达到20个。

民生保障持续改善。民生投入占地方财政支出的比重达到64.5%，14件为民办实事重点项目全面完成。城市居民人均可支配收入和农民人均纯收入分别增长9.8%和11.8%。新增城镇就业5万人，农村劳动力转移就业2.76万人，城镇登记失业率控制在2%。设立4000万元专项资金扶持创业带动就业，发放创业小额担保贷款6亿元，受益5180人，带动就业2.86万人。上调了城乡居民基础养老金、企退人员基本养老金、失业保险金、城乡最低生活保障标准及居民医保和基本公共卫生服务补助标准，提高了基层干部和教师工资待遇。放宽住房公积金贷款最高限额和年限，发放公积金贷款12.6亿元。建成保障性住房8460套，改造农村危房3318户。购置新能源公交车150辆。立体化治安防控体系逐步完善，安全生产形势稳定向好，食品药品综合监管得到加强。

社会事业协调推进。扎实开展创建全国文明城市活动，市民思想道德素质和城市文明水平进一步提高。现代公共文化服务体系不断完善，高校文体设施免费向社会开放，“一刻钟”公共文化服务圈覆盖率达到97%。成功举办了沂蒙画派晋京展。新建中小学和幼儿园175处，创建国家级重点中等职业学校5所。我市再次荣膺“国家卫生城市”称号。成功承办第23届省运会帆船帆板比赛，参赛省运会创历史最好成绩。人口和计划生育责任指标圆满完成。国防动员、双拥共建、人民防空和民兵预备役建设持续加强，军民融合深度发展。我市成功创建为全国社会组织建设创新示范区。支援新疆麦盖提县和扶贫协作重庆黔江区工作顺利推进。妇女儿童、青少年、残疾人、老龄、红十字等事业全面发展，民族宗教、打击走私、司法仲裁、国家安全、城管和文化市场执法、史志、档案、气象、水文等工作取得新成绩。

政府作风呈现新气象。扎实开展党的群众路线教育实践活动，重新拿起批评与自我批评的武器，深入查摆和整治“四风”问题，党员干部普遍经受了一次严格的党性教育和锻炼。坚持边查边改，认真落实市级20项问题整改清单，主动接受社会监督。严格落实党风廉政建设责任制，深入推进正风肃纪，查处违反中央八项规定精神问题182起、处理231人，对9起典型案件进行了公开曝光；查处“庸懒散”问题86件、处理96人，各级干部敬畏法纪、守规矩的意识明显增强。全面推进法治政府建设，自觉坚持依法行政，建立了政府法律顾问制度。主动接受人大法律监督和政协民主监督，认真做好人大代表建议、政协提案办理工作，办复率、满意和基本满意率均达到100%。扎实推进阳光政府建设，市长公开电话实现24小时在线值班，行风在线、网络问政、社情民意调查等民意诉求平台进一步畅通。

各位代表！

在经济下行压力持续加大的情况下，过去一年取得的成绩来之不易，凝聚着全市上下的心血和汗水。在此，我代表市人民政府，向辛勤工作在各条战线上的全市人民，向各级人大代表、政协委员，向各民主党派、工商联、无党派人士、各人民团体，向离退休老领导、老同志，向驻日照人民解放军和武警官兵，向所有关心支持日照建设与发展的社会各界人士和海内外朋友，表示衷心的感谢并致以崇高的敬意！

在肯定成绩的同时，我们也清醒地认识到面临的挑战和问题：经济发展进入新常态，下行压力持续加大，资源环境约束日益趋紧，新旧增长动力转换不快，对保持经济持续增长带来新挑战；综合实力不强、县域经济薄弱、高新技术产业和现代服务业比重偏低、创新和人才支撑不足等“短板”尚未得到根本解决；群众对教育、医疗、就业、社会保障、生态环境、安全生产等方面的问题反映仍较突出；面对新的形势和要求，有的干部在精神状态、行政方式、工作方法等方面，还存在一些不适应的问题等。对此，我们一定高度重视，扎实作为，努力解决。

二、2015年政府工作的总体思路和主要目标

今年是全面完成“十二五”规划的收官之年、全面深化改革的关键之年、全面推进依法治国的开局之年。全市经济社会发展的总体要求是：深入贯彻党的十八大及十八届三中、四中全会和习近平总书记系列重要讲话精神，坚持稳中求进、改革创新工作总基调，主动适应和引领新常态，以提高经济发展质量和效益为中心，着力解放思想、锐意进取，着力用好“蓝红金”和“一带一路”国家战略机遇，着力稳增长、转调创、强蓝区、促改革、惠民生、保稳定，努力推动全市率先创新优质发展，为实现“一个率先、五个日照”的奋斗目标奠定坚实基础。

主要预期目标是：生产总值增长9%左右，公共财政预算收入增长10%左右；固定资产投资增长15%左右，社会消费品零售总额增长12.5%左右，进出口总值与去年持平；规模以上工业增加值增长10.5%左右；城乡居民人均可支配收入分别增长9.5%和10%左右；新增城镇就业2.6万人、农村劳动力转移就业2万人。居民消费价格涨幅控制在3%左右；全面完成节能减排约束性目标。

各位代表！

当前我国经济发展已经进入新常态，认识新常态、适应新常态、引领新常态，是当前和今后一个时期经济发展的大逻辑。我们必须全面贯彻落实习近平总书记视察山东重要讲话和批示精神，按照省委确定的“一个定位、三个提升”的要求，深入开展“解放思想求创新、转型升级促发展”活动，坚定不移推动工作指导转变。必须始终把改革作为强大动力，不断释放改革红利，激发发展活力。必须坚持市场要活、创新要实、政策要宽，让每一个创业者都有如鱼得水的环境，形成大众创业、万众创新的生动局面。必须正确处理当前和长远的关系，既抓长远谋划，精心编制好“十三五”规划，更要干好当前，一年接着一年干，积跬步致千里，让日照的山山水水一天天变美变靓，让老百姓的生活一步步奔向全面小康！

三、全力推动新常态下的经济发展

加快推动结构调整和转型升级。逐个行业制定落实转型升级方案，推动现有产业向中高端迈进，大力引进培育产业链延伸项目，实现优势产业集聚集群发展。加快推进现代威亚30万台发动机增产、派沃泰自动变速箱三期、双港200万只高档活塞、岱摩斯车桥二期等重点项目建设，积极引进关联配套企业，培育壮大汽车及核心部件产业集群。日照钢铁精品基地年内力争完成投资89亿元，抓好宝华新材料二期、万方板业冷轧板、海汇海洋环保机械等钢铁深加工项目和国家碳素结构钢产品质检中心建设，推动钢铁产业向高端高质高效跨越。加快推进100个重点工业项目建设，加大对60家重点骨干企业扶持力度，实施251项重点技改项目，促进69个工业新增长点尽快发力。加大新兴产业培育力度，增强新兴服务业的支撑作用。加快推进星河科技园、文化创意产业园等平台建设，促进电子商务、文化创意等产业发展。依托日照大宗商品交易中心，积极开展大宗商品交易试点。大力扶持集装箱运输，支持商贸物流园区和仓储企业转型升级，加快发展现代物流业。搞好水上公园游客集散中心等旅游服务设施建设，积极推进海上垂钓、旅游小镇等项目，引导扶持提升乡村旅游，提高旅游业发展水平。加快发展养老、医疗、健康、体育、休闲、家政服务等生活性服务业和研发设计、金融保险、服务外包、市场营销等生产性服务业，积极培植高端装备、新材料、海洋生物、物联网、集成电路、新能源汽车、航空等新兴产业。大力实施便利消费、安全消费工程，完善同城物流配送网络，开展农超对接和社区生鲜配送，抓好公益性市场和平价超市建设。

大力培育民营经济。牢固树立与企业同舟共济的理念，重视发挥企业家作用，积极帮助解决融资、土地、市场等困难，努力形成大企业“顶天立地”、中小微企业“铺天盖地”的发展局面。认真落实市委、市政府《关于大力促进全市民营经济加快发展的意见》，加强政银企合作，将“财政增信优惠贷”规模扩大到30亿元，受益企业达到1000家以上。落实各项定向减税和普遍性降费政策，将财政对小微企业的补贴券规模扩大到300万元。通过政府补贴等办法在各类经济园区建设标准厂房，面向小微企业低价出租。加强中小企业公共服务体系建设，积极推动“个改小、小改规、规改股、股上市”，引导民营企业建立现代企业制度。

加强农业现代化建设。坚持“三个导向”，加快转变农业发展方式，提高农业综合生产能力和效益，持续增加农民收入。积极开展高产创建活动，稳定粮食总产。深入实施茶叶、桑蚕、烟叶、蜂业、中药材5个特色产业发展规划，提高市场竞争力和综合效益。加快农产品保鲜物流体系建设，提升农业龙头企业发展水平，实现农产品生产、加工、销售紧密衔接。完善农民合作社示范社、家庭农场示范场创建办法，强化工商资本进入农业的监管措施，做实新型农业经营体系。建设淘宝网“特色中国日照馆”，推动农产品流通电商化。扎实推进现代农业示范区、生态农业与农村新能源示范县建设，大力推行标准化清洁生产。针对去年以来持续发展的旱情，切实加强农田水利基本建设，做好农业抗旱应急工作，确保人畜吃水。

努力打造对外开放新优势。充分发挥日照作为新亚欧大陆桥经济走廊主要节点城市的作用，积极参与“一带一路”建设，着力打造“五通”示范区。大力推进中亚、中蒙通道建设，争取蒙古等国家在我市设立东方出海口和物流基地。做好第三届中国—中亚合作论坛承办工作，扩大我市在“一带一路”国家和地区的影响。抢抓中韩自贸区建设机遇，力争在我市设立国家层面的中韩产业园和自贸试验区。争取国家批准我市扩大口岸开放，继续加大综合保税区申报争办力度。坚持不懈地抓好招商引资工作，创新招商方式，由注重提供优惠政策向搭建创业平台转变，由注重建设园区向创造与国际接轨的工作生活环境转变。深化与现代汽车、金鹰、华润等大企业的合作，跟进落实我市在“全国知名民营企业入鲁助推转调创投资洽谈会”期间签约的56个项目。抓住大宗商品价格走低的有利时机，稳定能源资源性商品和关键零部件进口。积极鼓励民营企业走出国门，参与境外投资，加快培育本土跨国企业。推动日照银行引进境外战略投资者，加快窝窝商城在美国纳斯达克、华慧食品在马来西亚上市步伐。

推动区域经济协调发展。扶持激励区县走各具特色的发展路子，加快发展县域经济，支持东港区开展县域经济科学发展转型升级试点。加大扩权强镇力度，搞好夏庄镇行政管理体制综合改革试点。扎实推进基础设施和公共服务向农村延伸，启动实施青赵线改建工程，争取启动潮石路拓宽改造；推进城镇基本公共服务常住人口全覆盖。投资8000万元启动农村公路大修工程，年内完成200公里改造任务。投资3000万元，新建236个村的自来水工程，实现所有行政村通自来水。建设改造电气化村801个，全面消除农村电网低电压现象。

四、锲而不舍抓好打基础、利长远的大事

加快推进重大基础设施建设。对今年确定的100个市级大项目，建立强有力的协调推进、要素保障和督促考核机制，确保完成投资430亿元以上。（1）抓好机场飞行区罩面及航管楼、气象雷达站等配套工程建设，同步推进奎山综合交通枢纽、机场高速等工程，扎实做好航线开发、口岸通关等工作，确保机场年底前通航。（2）加快实施石臼港区规划调整方案，抓好30万吨级原油码头、30万吨级矿石码头、日照钢铁精品基地配套码头等重点工程建设。（3）开工建设潍日、岚罗高速公路，加快建设青日连铁路，争取早日开工青日连铁路与济青高铁联络线、日照至菏泽城际铁路，完成613省道西延一期和222省道西湖至岚山段拓宽改造工程。（4）抓好水网规划建设，“沭水东调”工程年内全线贯通，争取实施从连云港向岚山应急调水工程，开工建设日照水库扩容增效和户部岭水库除险加固工程。（5）开工建设220千伏五莲潮河、莒县源河等14项输变电工程。

加大生态环境保护力度。保护日照“蓝天碧海金沙滩”的环境优势，是全市人民的共同心愿。今年重点实施“六项生态工程”：（1）生态企业及片区建设。对钢铁、浆纸、化工、建材等重点行业和重点企业，实行逐步加严的环保标准，逐一明确污染治理的路线图、任务书、时间表和责任状。切实抓好日照港区、日照钢铁及周边等重点片区综合整治。（2）生态海洋建设。制定海岸带保护管理办法，严格保护岸线、沙滩、礁石、海岛、湿地、生物等海洋资源，抓好市区到森林公园段自然岸线的生态恢复。（3）生态流域建设。全面推行“河长制”，抓好崮河、沭河、营子河、后楼河等河流综合整治，分期分批开展小河小溪治理。（4）生态山体建设。严格划定禁采区，实施破损山体恢复治理工程，启动卧龙山公园保护整治。（5）生态走廊建设。抓好沿路、沿海、沿河植绿护绿，完成植树造林8万亩，争创国家森林城市。（6）生态乡村建设。深入推进美丽文明乡村行动，再投入4500万元完善城乡环卫一体化建设，提升村镇生态建设水平。

抓好城建重点项目。（1）加快城市总体规划（2013—2030年）和城市片区控制性详规编制工作，逐步实现经济社会发展规划、城乡规划、土地利用规划、生态环境保护规划“多规合一”。（2）加快华润中心城市综合体、钢铁大厦等项目建设，建成淄博路及地下工程。新增城市绿化面积211万平方米。（3）建成启用市图书馆，开工建设丁肇中科学馆。（4）加快智能环保公交系统建设，新购置50辆新能源公交车，启动城区公共自行车系统建设。（5）完成城中村改造房屋拆迁1200户，建设安置房50万平方米。

五、以更大力度推进改革创新

进一步简政放权。围绕营造国际化、法治化的营商环境，进一步转变政府职能，在公布实施行政审批事项目录清单的基础上，加紧制定推行行政权力清单、政府责任清单和市场准入负面清单，健全完善政务服务平台。再取消和下放一批行政审批事项，大力推行一口受理、限时办理、规范办理、透明办理、网上办理，坚决查处“中梗阻”，深入治理“审批难”，为企业和百姓办事创业营造更加宽松便捷的环境。深化商事制度改革，落实好事中事后监管责任。加快事业单位分类改革，继续推进事业单位法人治理结构建设试点。

推进农村综合改革。加强对农村土地承包经营权、房屋所有权确权登记颁证工作的质量管控，确保一次性把工作做扎实。以城中村、城郊村、乡镇驻地村、经济强村为重点，加快推进农村集体经济组织股份制改革。在区县普遍设立综合产权交易中心，搭建农村产权交易平台。探索土地承包经营权流转、抵押、担保等办法，推动农业适度规模经营。进一步深化供销社和水利改革。

深化财税体制改革。认真实施新预算法，构建全面规范、公开透明的预算制度。调整优化财政支出结构，大力压减投向一般竞争性领域的资金和“三公”经费支出，集中财力保基本公共服务、保民生、保改革。减少和取消财政对产业、企业的直接补助，采取基金、以奖代补、政府购买服务、政府与社会资本合作等多种形式，提高财政资金使用效率。完成机关和参公事业单位公务用车改革。对政府债务实行规模控制和预算管理。

加快投融资改革。积极引导企业利用多层次资本市场，到主板、中小板、创业板、“新三板”市场、海外资本市场及区域性股权交易市场募集资金。大力发展私募市场，推动政府引导基金的组建设立和规范运作，拓宽实体经济融资渠道。按照“非禁即入”、“非禁即准”原则，支持民间资本进入基础产业和基础设施、市政公用事业和保障性住房、金融服务等领域。完善社会征信体系，建设“信用日照”。全面落实监管责任，有效防范和化解金融风险。

提高创新驱动能力。把人才培养和引进放在突出位置，制定让人才在创新成果运用中有份额、有股权的政策措施，让做出创新贡献者“名利双收”。用好中国蓝色经济引智试验区机制，整合各类引智资金，增强人才支撑效果。发挥大学科技园作用，进一步完善大学生创新创业平台。强化企业在技术创新中的主体地位，新建市级以上企业技术中心19家、工程技术研究中心15家。加大对水产研究所、茶科所等重点科研院所的扶持力度。抓好“云计算数据中心及云计算产业基地”项目，加快建设智慧城市。

六、扎实办好民生实事

完善就业创业和社会保障体系。继续实施大学生就业促进计划和创业引领计划，建设市级公共创业孵化基地，设立创业大学。全面实施农民工职业技能提升、权益保障、公共服务3项三年行动，建设市级农民工综合服务中心。将城乡居民基础养老金最低标准提高到每人每月85元；城市低保标准提高到每人每月460元，农村低保标准提高到每人每年3100元；城乡居民医保财政补助标准提高到每人每年380元；基本公共卫生服务经费财政补助标准提高到人均40元；大病保险年度最高补偿限额提高到30万元。继续提高企退人员基本养老金、失业保险金待遇水平。继续做好符合条件的被征地农民参保缴费工作。实施机关事业单位养老保险制度改革。开展长期医疗护理保险试点。为农村妇女免费开展“两癌”筛查，为符合生育政策的夫妇免费提供孕前检查，为65岁以上老年人免费进行健康查体，为1000名贫困白内障患者免费实施复明手术。开工建设保障性住房11400套，新增租赁补贴400户，改造农村危房2000户。建成启用市社会福利中心老年养护楼。健全精准扶贫机制，帮助1.4万农村贫困人口脱贫。

全面发展社会事业。坚持文化惠民、文明育心，大力实施社会主义核心价值观载体化工程，深入开展文明创建活动，打造“德润港城、善行日照”品牌。深入实施“千百重点文化工程”，加快构建现代公共文化服务体系。加强文化遗产和文物保护。搞好文艺精品创作。办好第五届市运会。坚持教育优先发展，大力推动义务教育薄弱学校改造，确保各区县通过国家义务教育发展基本均衡县评估验收。完成市特殊教育中心建设，新建60个公益性幼儿园。加强教师队伍建设，提高教育教学质量。完善现代职业教育体系，大力培养职业技术人才。加快推进公立医院综合改革。稳步推进医药价格改革，巩固完善基本药物制度改革成果。稳定适度低生育水平，加大出生人口性别比综合治理力度，提高出生人口素质。加强国防动员实战化建设，落实优抚安置政策，促进军民融合发展，争创全国双拥模范城“五连冠”。继续做好援疆和扶贫协作黔江区工作。

深入推进“平安日照”建设。全面推进依法治市，大力弘扬社会主义法治精神，提高全民法治意识。加大社会治理创新力度，扎实做好新形势下的信访工作，完善人民调解和社区矫正工作机制，加强矛盾纠纷排查化解。健全完善全方位、全天候、全员化的维稳体系和立体化治安防控体系，强化社会治安综合治理，严厉打击各类违法犯罪。抓好刑罚执行示范区建设。积极推广仲裁制度，依法管理宗教事务，严厉打击走私贩私，深化社会组织登记管理体制改革。强化食品药品监管体系建设，实施“清洁厨房”工程，确保食品药品安全。牢固树立安全生产“红线意识”，严格落实企业主体责任、政府部门监管责任和属地管理责任，深入排查治理安全隐患，完善应急管理机制，确保人民群众生命财产安全。

七、切实加强政府自身建设

全面落实依法治国、从严治党新要求，努力践行为民务实清廉，不断巩固拓展党的群众路线教育实践活动成果，适应新常态，展现新作为。

依法履行政府职能。各级政府工作人员特别是领导干部必须带头学法、尊法、守法、用法，坚持“法无授权不可为、法定职责必须为”，严格按照法定权限和程序行使权力、履行职责。健全依法科学民主决策机制，严格规范行政决策程序，建立重大决策终身责任追究制度及责任倒查机制。完善政府法律顾问制度，加强和改进行政复议、行政应诉工作。建立领导干部干预司法活动、插手具体案件处理的记录、通报和责任追究制度，坚决杜绝以言代法、以权压法、徇私枉法。认真落实人大及其常委会各项决议决定，自觉接受人大法律监督和政协民主监督，认真听取各民主党派、工商联、无党派人士、各人民团体和人民群众的意见，主动接受司法监督、新闻舆论监督和社会公众监督。

努力提高能力素质。新的形势和任务，对各级领导干部的能力素质提出了新的挑战。各级政府必须大兴学习调研之风，学习新知识，掌握新本领，适应新常态。深入学习党的十八大及十八届三中、四中全会和习近平总书记系列重要讲话精神，自觉用中国特色社会主义理论体系武装头脑、指导工作。加强对宪法法律的学习，破除传统思维定势和行政方式，学会运用法治思维、法治程序、法治方式来深化改革、推动发展、维护稳定。加强调查研究，积极探索新常态下的经济社会发展规律，学习新常态下的领导方法，提高引领新常态的能力。

坚持不懈反“四风”。扎实开展“三严三实”专题教育活动，引导各级干部不断转变作风，切实做到心中有党、心中有民、心中有责、心中有戒。持续推进中央八项规定精神的贯彻落实，坚持政府带头过紧日子，从严控制公用经费支出，确保财政供养人员和“三公”经费只减不增。持之以恒正风肃纪，紧盯“四风”新动向、新变种，加大明察暗访力度，坚决防止“四风”反弹。牢固树立权力就是责任的意识，大力弘扬勇于担当精神，既要守规矩又要干事业，坚决纠正为官不为、懒政怠政。

加强政府廉政建设。政府领导班子成员必须严格履行党建责任和党风廉政建设责任制，带头守纪律讲规矩，带头执行中央八项规定，带头遵守廉洁从政各项规定，管好自己，带好队伍。健全行政审批、公共资源交易、国有企业监管等制度，完善防止权力寻租和利益输送的体制机制，把权力关进法纪制度的“笼子”。强化监察、审计监督和政府内部层级监督，完善问责机制。坚持对腐败行为零容忍，有腐必反，有贪必肃，始终保持高压态势，决不让腐败分子逍遥法外。

各位代表！

顺应新常态，开启新征程，是时代赋予我们的历史使命。让我们紧密团结在以习近平同志为总书记的党中央周围，高举中国特色社会主义伟大旗帜，在中共日照市委的坚强领导下，进一步解放思想，锐意进取，齐心协力，真抓实干，不断开创经济社会发展新局面，为谱写全市人民美好生活新篇章而努力奋斗！
